# Serosurvey of veterinary conference participants for evidence of zoonotic exposure to canine norovirus – study protocol

**DOI:** 10.1186/1743-422X-9-250

**Published:** 2012-10-30

**Authors:** João Rodrigo Mesquita, Maria São José Nascimento

**Affiliations:** 1 Laboratório de Microbiologia, Departamento de Ciências Biológicas, Universidade do Porto, Rua Jorge Viterbo Ferreira, 228, 4050-313, Porto, Portugal; 2 Secção de Ciências Veterinárias, Instituto Politécnico de Viseu, Quinta da Alagoa - Estrada de Nelas, Ranhados, 3500-606, Viseu, Portugal

**Keywords:** Canine norovirus, Occupational exposure, Zoonosis, Veterinarians, Public Health, Risk factors

## Abstract

**Background:**

Noroviruses have emerged as the leading cause of outbreaks and sporadic cases of acute gastroenteritis in humans worldwide. Person-to-person contact and consumption of contaminated food are considered the most important ways of transmission of noroviruses however zoonotic transmission has been suggested. Recently, noroviruses have been found in dogs which, unlike bovine and swine noroviruses, may present a higher risk of zoonotic transfer, given to the often close contacts between humans and pet dogs in many societies across the world. The present paper describes a seroepidemiologic study aiming to provide information on the exposure level of humans to canine norovirus.

**Methods/Design:**

A case–control study was designed to address the potential exposure to canine norovirus based on the presence of antibodies against canine norovirus. Sera from veterinarians (a population repeatedly in close contact with dogs) will be collected in an annual Veterinary Sciences Congress in Portugal. In addition, sera from general population will be obtained and used as controls for comparative purposes. All sera will be tested for the presence of canine norovirus antibodies using a virus-like particle-based enzyme immune assay. Risk factors for canine norovirus antibodies presence in veterinarians will be investigated through the delivery of an anonymized questionnaire to the participants.

**Discussion:**

The present study aims to identify seropositive individuals to canine norovirus and to assess risk profiles among veterinary professionals with occupational exposure to dogs. To our knowledge this is the first study providing information on the potential zoonotic risk of canine norovirus, thus allowing the development of preventive measures and ascertaining potential risks for Public Health resulting from contact to dogs.

## Background

Noroviruses are the leading cause of outbreaks and sporadic cases of acute gastroenteritis worldwide in humans [1]. Person-to-person contact and consumption of contaminated food are the most important routes of transmission [2,3], however zoonotic transmission has been recently suggested [4,5].

Both humans and animals shed genetically similar norovirus strains which raises the questions whether transmission of these viruses between animals and man and vice versa occurs and whether animals represent a reservoir for noroviruses [6]. In 2005, researchers have sought explicit criteria to judge the possibility of animal infections causing human diseases by performing a qualitative risk assessment of the emerging zoonotic potential of animal diseases [7]. According to the proposed algorithm animal noroviruses were categorized as not zoonotic (grade 0 in a scale of 0 to 4) [7]. However, additional research has strengthened the hypothesis for zoonotic transmission primarily based on the genetic relatedness between human noroviruses and noroviruses found in swine [4] and the experimental confirmation of human norovirus replication in gnotobiotic pigs that also presented diarrhea and fecal viral shedding [5]. Additionally, human norovirus sequences were found to be present in retail meat samples alerting for a possible route for zoonotic transmission of noroviruses through the food chain [8]. Results from these investigations have raised alarm for norovirus cross-species transmission to humans through infected domestic animals, and for the emergence of animal/human recombinants [8].

Recently, noroviruses have been described in dogs from Portugal [9], Italy [10] and Greece [11]. Unlike bovine and swine noroviruses, canine norovirus could represent a higher risk of zoonotic transfer, given the intimate interaction between humans and pet dogs in societies worldwide. Interestingly, researchers from Finland have found that dogs could shed human norovirus therefore posing as potential carriers for human norovirus [12]. Although severe cases are uncommon, zoonotic agents that are transmitted due to intimate contact between pets and humans are widely documented [13], specially by dogs which are known to play a role in about 100 zoonotic diseases [14,15].

The anthropozoonotic ability of an infectious agent has been long inferred by studies identifying specific antibodies against a pathogen in a human population that is repeatedly in close contact with a particular animal and comparison to the antibody level of a matched control population with no animal association [16].

To the best of our knowledge, since the discovery of canine norovirus in 2008 [10] no project was approved or research has been made worldwide concerning the study of the potential zoonotic exposure to canine norovirus. Moreover, the potential for human exposure to canine norovirus is great, since it has been shown that noroviruses are widely shed by dogs [17,18].

The project NOVEL CANINE NOROVIRUS: MOLECULAR, EPIDEMIOLOGIC AND PATHOGENIC ASPECTS, is supported by both European and Portuguese funding and aims to answer this question by providing for the first time detailed information on the exposure level of humans to canine norovirus through the evaluation of the presence of antibodies to canine norovirus in human sera in a large-scale case–control epidemiologic survey. Moreover will aim to compare the serological responses between two kinds of populations with high and low-level canine norovirus exposure risk profiles, respectively small animal veterinarians (that frequently encounter occupational exposures to animal material that place them at high risk for zoonoses) and general population (with no professional exposure to animals but potentially exposed to household pets). Finally, the study will investigate the potential risk factors for canine norovirus exposure among veterinary workers. Overall, this work will address the need for the development of preventive measures against this potentially new zoonotic agent such as viral control programmes, and the subsequent need for vaccine research and development.

## Methods/design

### Location and population

Sera from veterinarians will be collected in Santa Maria da Feira, northern Portugal, at a Veterinary Sciences Congress (VSC) which occurs each year in February. The VSC is mainly attended by Portuguese veterinarians, and is generally considered the congress within the Veterinary Sciences with the highest number of participants in Portugal. The first VSC started in 2005 with 350 participants, the second and third VSC (2006 and 2007) both had 550 participants, the fourth VSC (2008) had 750 participants, the fifth VSC (2009) had 1000 participants, the sixth VSC (2010) had 1200 participants, the seventh VSC (2011) had 1300 participants. The growth of the number of participants in the past years provides confidence for a substantial number of congress participants willing to participate in our study. Blood (~5ml) will be collected by a group of trained nurses by venepuncture. Sera will be separated and frozen at −20°C within 24 hours. Participants will be asked to provide for signed informed consent in accordance to the Helsinki Declaration. In order to achieve the highest number of participants for this study, posters will be fixated and flyers will be introduced in the participants’ documentation bag.

For comparative purposes, sera from general population will also be studied. These sera will be obtained from volunteers at the Faculty of Pharmacy of the University of Porto and will include students, technical staff and Professors. To reduce possible confounding effects these control sera will be matched by age (5-year age group), sex and district. The number of sera samples from the general population will be estimated through statistical inference and the validity of the control group will be proven by a χ2 test for unequal odds with Yates’ continuity correction. This test will assess if the differences of seroprevalence of antibodies to canine norovirus among veterinarians and the general population will be statistically significant therefore confirming that the control sera will be valid comparison group for sera from veterinarians.

This study was approved by and ethical commission at the University of Porto.

### Structured questionnaire

Participants will be asked to complete an anonymized structured questionnaire that will include information about variables that are considered potentially associated to zoonotic infections such as professional details (years in practice, history of needle stick injury with dog blood and/or other animal’s blood) and personal details (household dog/other animal during childhood), as well as demographics (age, sex, and district of residence). To maintain the anonymity, each serum sample and questionnaire belonging to the same participant will be given the same unique number.

### Assessment of risk factors for antibody to canine norovirus seropositivity among veterinarians

In order to characterize putative risk factors for canine norovirus antibody seropositivity in veterinarians, univariate and multivariate logistic regression analysis will be performed. Multivariate models will be chosen because are most informative given that they produce adjusted odds ratios (aOR) that simultaneously measure the strength of associations between the multiple risk factors and the canine norovirus serum antibody presence. Crude odds ratio (cOR) and aOR will be calculated to assess the presence of confounding factors. Variables will be considered independent when the calculated cOR and aOR are similar. Likelihood ratio test will also be performed to evaluate statistical significance of risk factors with more than 2 levels, given its independence from the variables reference level. All analyses will be performed using Epicalc package in the R software (R 2.1.2.0) (R Development Core Team, 2010).

### Laboratory analysis

Immunoglobulin (Ig) G antibodies to canine norovirus will be detected by an in-house enzyme immune assay using recombinant virus-like particles (VLPs) of canine norovirus as antigens. VLPs will be produced in a baculovirus-insect cell expression system and purified through sucrose and CsCl gradients as described elsewhere [19]. VLP morphology will be confirmed by electron microscopy. The antigenicity of VLPs will be tested by western blot analysis using rabbit hyperimmune antisera against canine norovirus. A checkerboard titration of canine norovirus VLPs, human sera, and conjugate (goat anti-human IgG horseradish peroxidase) will be performed to determine the optimum conditions of the assay. Sera from both veterinarians and population controls will be tested in duplicate.

### Evaluation of cross-reactivity

To investigate the possibility of sera cross-reactivity all the sera will be tested for the presence of antibodies to human norovirus by an enzyme immune assay. Crossed-reactions between canine norovirus and human norovirus antibodies will be evaluated using a correlation test calculated with a 95& significance level, after plotting data from optical densities in histograms. If optical densities are normally distributed, a Pearson’s correlation test will be used. If optical densities are not normally distributed, a Spearman’s correlation test will be chosen. Analyses will be performed using GraphPad Prism ver. 2.01 software (GraphPad Software, San Diego, CA).

## Discussion

In the present study we hypothesize that the outcomes of the proposed serosurvey may provide unique insight in the potential exposure of humans to canine norovirus. In addition, different risk profiles between veterinary professionals (with occupational contact to dogs) and the general population will be evaluated. This will provide valuable data on the potential zoonotic risks of canine norovirus for humans.

Among veterinary workers, potential risk factors for canine norovirus infection will also be assessed. This study will provide important data to support possible professional/occupational adjustments if needed, to decrease risk for exposure to canine norovirus. Finally, this study will provide more data on the zoonotic exposure risk and potential preventive measures, such as canine norovirus control programmes (e.g. quarantines), and canine norovirus vaccine research and development. It is also expected to disseminate the results to both public health (by communicating in National and International Conferences) and academic stakeholders by publication of the results in peer-reviewed journals.

Epidemiologic studies studying the norovirus human-animal interface are rare and the proposed study on canine norovirus will attempt to fill this knowledge gap.

## Competing interests

The authors declare that they have no competing interests.

## Authors’ contributions

JRM and MSJN equally contributed to the design of the study. Both authors, drafted, revised and approved the final manuscript.
